# ROS 4 healthcare: a framework for physiological human sensing for social, assistive, rehabilitation, and medical robotics

**DOI:** 10.3389/frobt.2026.1745197

**Published:** 2026-04-07

**Authors:** Ricardo Javier Manríquez-Cisterna, Pranjal Mishra, Jorge Peña-Queralta, Monica Perez-Serrano, Spyridon Garyfallidis, Lucas Kupper, Mehdi Ejtehadi, Alexander Breuss, Ankit A. Ravankar, Jose Victorio Salazar Luces, Robert Riener, Yasuhisa Hirata, Diego Paez-Granados

**Affiliations:** 1 Smart Robots Design Lab, Department of Robotics, Graduate School of Engineering, Tohoku University, Sendai, Miyagi, Japan; 2 Spinal Cord Injury and Artificial Intelligence Lab, Department of Health Science and Technology, ETH Zurich, Zurich, Switzerland; 3 Digital Health Care and Rehabilitation, Swiss Paraplegic Research, Nottwil, Switzerland; 4 Sensory-Motor Systems Lab, Institute of Robotics and Intelligent Systems, ETH Zurich, Zurich, Switzerland; 5 Spinal Cord Injury Center, University Hospital Balgrist, Zurich, Switzerland

**Keywords:** biosignals, healthcare robotics, human-robot interaction, medical robotics, open-source, rehabilitation robotics, robot operating system, social robotics

## Abstract

The pervasive integration of robots into daily life necessitates advanced human-robot interaction (HRI) capabilities, particularly the accurate understanding of human physiological and cognitive states. The current state of the widely used Robot Operating System (ROS2) lacks standardized mechanisms for representing and communicating human states. This paper introduces ROS 4 Healthcare (ROS4HC), a comprehensive open-source framework designed to standardize the acquisition, representation, and integration of human sensing data into robotic systems. ROS4HC provides unified message types, modular sensor drivers, signal processing libraries, and visualization tools for physiological, biological, and physical signals. This framework is validated through empirical case studies in healthcare robotics, including a heart rate (HR)-adaptive wheelchair velocity modulation, an autonomous treadmill system integrating physiological feedback, and a nocturnal monitoring system based on a robotic rocking bed. These case studies demonstrate that the framework enables modular component reuse, standardized communication, and interoperability for better human-robot integration. Beyond healthcare, we highlight ROS4HC’s generalizability for critical applications such as industrial safety, human-robot collaboration, and performance monitoring, establishing a standardized infrastructure for safer, more adaptive, and context-aware robotic systems across diverse domains.

## 1 Introduction

Robotics is a field with broad applications. In recent years, we have seen the advancement of robotics seep into different aspects of our daily lives, like robot vacuums Frennert and Östlund (2015), self-driving drones Gaber et al. (2021); Aliane (2024), Advanced Driver-Assistance Systems (ADAS) for automobiles Brijs et al. (2024); Louis et al. (2022), AI conversational agents Li et al. (2025); Norris et al. (2025), and others. With current advancements in technology, we expect these systems to be ubiquitous, more useful, and autonomous. If we take into account traditional robotic systems such as mobile bases or manipulators, ROS2 Maruyama et al. (2016) serves a big task of abstracting different hardware and software into a unified framework allowing rapid development of robotic solutions. Good examples are LiDARs, cameras, IMUs, or on the software side, the navigation stack NAV2 Macenski et al. (2020) is a prime illustration. This kind of integration and modularization greatly accelerates robotics development and has given rise to multiple solutions which were unthinkable before.

**FIGURE 1:**
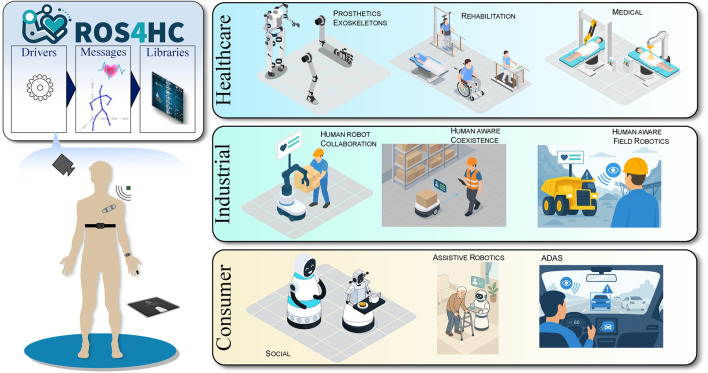
Illustration of ROS4HC framework components. With ROS4HC, we connect under the same umbrella wearables and other user sensors, robots, and AI algorithms aimed at healthcare monitoring. Visualization tools are tailored to either robotics/AI developers, or to clinicians or therapists.

Despite these advancements, a critical gap persists: robots that work closely with humans still struggle to accurately understand their operators or end-users. This limitation hinders the development of truly adaptive, safe, and personalized human-robot interactions Honig and Oron-Gilad (2018); Rodríguez-Guerra et al. (2021); Manríquez-Cisterna et al. (2025). To address this, we propose a new framework for human state data acquisition and processing for robotics which we have named ROS4HC. The objective is to present the human state for different robotic actors to interact with, enabling robots to respond intelligently to human physiological, biological, and physical signals. The framework components of ROS4HC are shown in Figure 1.

For this to be feasible, a standardization effort is required to bring human sensing into a unified framework with consistent message types and a robust infrastructure to implement new algorithms or libraries. This allows robotic systems to make effective use of this data when interacting with humans. We envision a future Kim et al. (2021) where, for example, human alertness can be quantified by installing a mmWave radar, a wearable Photoplethysmography (PPG) sensor, or even a camera. The main outcome is that other actors can build upon this framework and implement their own solutions without being experts in healthcare or human sensing. Applications of this kind are ubiquitous, from human monitoring on vehicles to prevent accidents, ADL classification, healthcare aware robots in rehabilitation, knowing that medical robots are a key area among the grand challenges of Science Robotics Yang et al. (2018), and others. Demographic challenges around the globe particularly in Europe and Asia have prioritized and accelerated the integration of AI and healthcare robotics Czaja and Ceruso (2022). For example, in Japan, where aging is a major challenge, the government has invested heavily into research and development of human-robot interaction initiatives through means of Japan’s Moonshot Projects, in particular, Goal 3^1^ which intends to standardize these interactions and develop robots that autonomously adapt and evolve alongside humans, as shown by Hirata et al. (2023) Ravankar et al. (2023). This work is also a direct consequence of one of the Moonshot Challenges.

Human–Robot Interaction is rapidly moving beyond laboratory environments into real-world applications in healthcare, rehabilitation, and collaborative industrial settings. The ability to perceive and respond to human physiological and behavioral states in real-time is a key enabler for adaptive and human-aware robots Kruse et al. (2013); Freire et al. (2024); Singamaneni et al. (2024). Wearable sensors, contactless modalities, and multimodal fusion approaches provide unprecedented opportunities to monitor cognitive workload Tamantini et al. (2025), mental fatigue, and affective states, yet integration into robotic pipelines remains fragmented and limited by the absence of standardized frameworks Kothe et al. (2025); Bohus et al. (2021).

Without consistent abstractions and interoperable software infrastructures, extensive application-specific integration is required, hindering reproducibility, scalability, and deployment beyond specialized research settings Mohamed and Lemaignan (2021); Jo et al. (2021). Standardization—both in software and data representation—is therefore critical for leveraging multimodal human sensing across diverse domains, from assistive rehabilitation to industrial collaboration Estrin and Sim (2010); IEEE (2021); Lasota et al. (2017); Ajoudani et al. (2018).

While healthcare and rehabilitation are primary application domains, the principles underlying ROS4HC extend to other contexts requiring human state awareness. Physiological signals such as heart rate and heart rate variability have been shown to reflect physical and cognitive load beyond clinical settings Solhjoo et al. (2019); Kodikara et al. (2025):

1. Industrial Safety: Monitoring fatigue or stress enables collaborative robots to adjust behavior and reduce accident risk in shared workspaces Li et al. (2023).
2. Human-Robot Collaboration: Adapting robot speed, force, or autonomy based on human exertion or attention improves efficiency and safety Bassi et al. (2025).
3. Sports and Performance Training: Quantifying physiological metrics like heart rate, breathing rate, and movement patterns to provide real-time feedback and optimize workload and reduce injury risk Mezzani et al. (2013); Sammito et al. (2024).

This paper details the design, implementation, and practical utility of ROS4HC. We present its core components, including standardized messages, device drivers, processing libraries, and visualization tools, highlighting how it simplifies the integration of diverse human sensing modalities.

We demonstrate the framework’s capabilities through real-world use cases in robotics and discuss its broader implications and generalizability for enhancing human-robot interaction across various safety-critical and performance-oriented applications, such as, stress-adaptive operation of an electric wheelchair, gamified robotic treadmill control and heart-rate-based rocking bed control. These scenarios showcase how understanding human physiological states can lead to more personalized and effective care solutions, as well as underline the necessity of integrating biosensors and wearables into robotic systems to address the challenges of future robotics.

### 1.1 State-of-the-art

Integrating physiological and multimodal human sensing into robotic systems has attracted increasing attention in recent years, particularly in the context of human–robot interaction (HRI), assistive robotics, and collaborative automation. Recent surveys Nascimento et al. (2020) highlight growing use of wearable and non-invasive physiological sensors such as; ECG, EDA, EMG, respiration, and EEG to infer affective, cognitive, and physical human states during interaction Savur and Sahin (2023); Liu and Liu (2023); Mu et al. (2025); Bussolan et al. (2025); Zhang et al. (2025); Farmani et al. (2025); Gkikas et al. (2025). However, despite rapid advances in sensing hardware and signal processing, the resulting software landscape remains fragmented across robotics, affective computing, and digital health.

Robotics middleware such as ROS Industrial Edwards and Lewis (2012) and Autoware Kato et al. (2018) have standardized perception, planning, and control in industrial and autonomous driving applications, yet they do not natively support physiological sensing or health-oriented human state representations. Consequently, applications relying on biosignals—such as affect and workload estimation Cittadini et al. (2023), activity-of-daily-living monitoring for rehabilitation Ejtehadi et al. (2023), or myoelectric and neuromuscular control Campbell et al. (2025)—typically employ custom, tightly coupled pipelines that limit reuse, interoperability, and reproducibility Jo et al. (2021).

Several frameworks address complementary aspects of this challenge. Lab Streaming Layer (LSL) remains a *de facto* standard for synchronizing heterogeneous physiological and behavioral sensor streams with high temporal precision Kothe et al. (2025). Its widespread adoption is further reflected in recent multimodal datasets for human–robot collaboration that rely on precise temporal alignment of biosignals Bussolan et al. (2025). Nevertheless, LSL is not designed for robotics: it lacks semantic representations of human state and does not integrate naturally with robot control architectures or ROS communication paradigms.

The Platform for Situated Intelligence (ψ) provides a powerful environment for building multimodal AI pipelines with time-aligned data fusion, visualization, and debugging Bohus et al. (2021). While recent work demonstrates its effectiveness for complex multimodal reasoning, ψ operates largely outside robotic middleware and does not address domain-specific physiological semantics, healthcare interoperability, or real-time robot control requirements.

Another framework introduced around the year 2011 for sensor integration is the Social Signal Interpretation or SSI Wagner et al. (2013), this framework aims to allow real-time recognition of social signals to provide intuitive and natural human-computer interaction. It is designed to be modular, integrate into machine learning workflows and has a C++ and Python API. It also solves the problem of synchronization but it only works on the Microsoft Windows® operating system. Taking into account the current requirements of robotics researchers, this framework does not fit the common development environments of robotics and is also geared more towards communication and not the understanding of the human state.

Within the Brain-Computer Interface (BCI) community another commonly used open-source framework is OpenViBE Renard et al. (2010), although it is only used for brain related modalities like EEG, MEG & EMG, there are efforts in the robotics community to interface it with ROS2 using ROS Neuro. Its high specificity for brain related modalities, makes this a very interesting framework but leaves modalities uncovered, which our proposed framework targets.

Also to note, Central Control Software (CCS) is a modular, open-source platform designed to act as a central hub for the seamless integration of heterogeneous hardware and software modules in human-centered research Tommaso et al. (2024). CCS is engineered for high-level closed-loop management and real-time interaction between diverse systems, such as wearable robots, physiological sensors, and augmented reality environments CCS provides an intuitive. NET-based GUI suitable for both technical and non-technical researchers. However, its current implementation as a Windows Form Application may present integration challenges for researchers operating strictly within Linux-based robotic middleware environments like ROS2.

In the ROS ecosystem, efforts toward human-centric representations are emerging. ROS4HRI proposes standardized models for social perception, enabling interoperable pipelines for face, body, voice, and interaction cues Mohamed and Lemaignan (2021). ROS Neuro Tonin et al. (2022) and related projects integrate EEG and EMG acquisition for brain–computer and neuromuscular interfaces, while recent ROS 2 wearable biosensor ecosystems facilitate streaming physiological data into HRI pipelines Jo et al. (2021). Although these approaches demonstrate the feasibility of physiological integration in ROS, they remain limited in semantic breadth, device coverage, or healthcare alignment, and do not provide a unified abstraction for physiological human state modeling Liu and Liu (2023); Mu et al. (2025).

Outside robotics, recent physiological computing toolkits and wearable platforms emphasize robust multimodal acquisition, signal quality monitoring, and real-time fusion Liu and Liu (2023). Similarly, emerging multimodal physiological datasets for collaborative robotics underscore the growing demand for synchronized, high-quality biosignal streams Bussolan et al. (2025). However, these systems are typically decoupled from robotic middleware, requiring ad-hoc adapters to interface with control loops.

In parallel, healthcare-oriented standards such as Open mHealth Estrin and Sim (2010), HL7 FHIR Health Level Seven International (2025), and IEEE 11073 ISO/IEEE (2024) define mature schemas and protocols for physiological and wearable data interoperability. Despite their widespread adoption in digital health, these standards are rarely implemented within robotic middleware, further reinforcing the disconnect between physiological sensing and robot control.

Overall, recent work addresses isolated dimensions of the problem—temporal synchronization Kothe et al. (2025), multimodal AI pipelines Bohus et al. (2021), wearable sensing Liu and Liu (2023), or social interaction semantics Mohamed and Lemaignan (2021). No existing framework provides a robotics-native, modality-agnostic, and healthcare-oriented infrastructure that unifies standardized semantics, temporal coordination, and integration with robot control. This persistent fragmentation continues to hinder scalability, reproducibility, and deployment of physiology-aware HRI systems, motivating the development of ROS4HC.

## 2 ROS4HC framework and architecture

In robotics, commonly used sensors such as LiDARs, IMUs or image sensors are well-integrated into the ROS ecosystem, streamlining the development of robot perception, localization and control. Integrating physiological data from medical devices and wearable sensors into robotic systems remains a non-trivial task, as these devices typically lack native ROS compatibility and necessitate extensive custom programming for data acquisition, synchronization, and integration. ROS4HC seeks to bridge this critical divide by providing a standardized framework tailored to seamlessly integrate medical device and wearable sensor data into ROS-based systems. Drawing inspiration from established design conventions proposed by ROS4HRI, ROS Neuro, ROS Industrial, and Autoware, ROS4HC aims to simplify the integration of health data and biosignals into robotic applications, providing a unified framework that facilitates reproducibility and accelerates research in the field.

### 2.1 Design principles

ROS4HC adopts the main ROS 2 design principles: distribution, abstraction, asynchrony, modularity, and hierarchical data management, to ensure robust, flexible, and scalable integration of health-related sensors in healthcare and general robotics. Its distributed architecture supports seamless data exchange across diverse systems, while abstraction standardizes interfaces, enabling sensor-agnostic application development. Asynchrony ensures effective handling of heterogeneous data streams for real-time monitoring and long-term analysis. Modularity fosters reusable, interoperable components, streamlining development and enhancing system resilience. Hierarchical data management organizes health data using logical naming conventions, promoting clarity, consistency, and interoperability across applications.

### 2.2 Design requirements

The design requirements for ROS4HC are:

1. Robot and Sensor Agnosticism: Ensure compatibility with a wide range of robotic platforms and sensor types, enabling flexible integration.
2. Time-Scale Independence: Support both real-time data processing and long-term data logging to cater to diverse needs.
3. Ease of Use: Prioritize intuitive interfaces and workflows for developers and end-users to simplify application development and deployment.
4. Extensibility and Inclusivity: Facilitate the addition of new devices, message types, and contributions from the community, promoting growth and collaboration.
5. Generalizability: Design the system to be applicable across various scenarios and domains beyond healthcare, ensuring versatility.

### 2.3 Component description

The ROS4HC framework is composed of four distinct pillars, as shown in Figure 1, each facilitating the integration of health-related data into the ROS ecosystem: Messages, Drivers, Libraries, and Utilities.

#### 2.3.1 Messages

Messages serve as the foundational communication units within the ROS4HC framework, encapsulating various health-related signals obtained from medical devices and wearables. These messages were designed to encode essential physiological parameters such as vital signs, biofeedback data, and other relevant health metrics into a standardized format, ensuring interoperability and ease of data exchange. We have defined a comprehensive set of message types across four main categories: Physiological, Physical, Monitoring Activity, and Hardware.

#### 2.3.2 Drivers

Drivers interface the specific medical devices and wearables to extract the data streams provided by the device and converts them into standardized ROS4HC messages. By leveraging standard protocols and APIs, they enable acquisition from a wide range of devices. This approach liberates researchers and developers from the burden of integration challenges, allowing them to focus on higher-level tasks such as algorithm development and application logic.

The actual data that can be extracted from the devices depends on the manufacturer, in some cases manufacturers will produce derived metrics and also the underlying raw data, in both cases ROS4HC strives to provide messages for both styles of data.

To clarify what raw data means in this context, HR can be used as an illustrative example. HR is typically measured indirectly, using techniques such as Photoplethysmography (PPG) or single-lead electrocardiography (ECG). In this setting, raw data would refer to the underlying sensor signals, such as optical intensity in the PPG sensor or the voltage time series of the ECG. Although these measures incorporate rich physiological information, their interpretation is context-dependent and generally not actionable in non-specialist settings. For this reason, algorithms use this raw data to obtain actionable measures, such as counting the heartbeats from the waveform to obtain HR. As a result, consumer devices will tend to report standardized, interpretable, and actionable measures, which are generally processed signals, instead of ECG or PPG waveforms, which are geared towards algorithm development or specialist analysis.

From our experience during sensor selection and software development we have come to the following classification, this makes it easier to understand what are the challenges and the abilities of each device

1. Direct Connection Sensors: These devices allow data access via an SDK and connect locally, typically through Bluetooth®. Depending on the device, data can either be:
  - Streamed in real-time, or
  - Stored locally (e.g., on a smartphone) for later download.
2. Cloud-Enabled Sensors: These devices store data on a server, which must be retrieved through a cloud-based solution.

ROS4HC is an open-source initiative, which strongly emphasizes community involvement. In the case of Direct Connection Sensors, the ROS4HC driver executes within the ROS2 environment, typically on a Linux PC. Communication with the device is direct, most commonly over Bluetooth®. The data path is defined by either vendor-provided device APIs, protocol documentation or through independent protocol implementations. The driver is responsible for handling device communication and translating the messages into the standardized ROS4HC message formats.

For Cloud-Enabled Sensors, the driver also runs in the ROS2 environment. But does not communicate with the device directly. Instead, it interfaces with a manufacturer-managed backend or cloud service where data is made available after device synchronization and in some cases, manufacturer-specific postprocessing. This may introduce additional external dependencies on vendor-provided cloud data access mechanisms, and may require subscription-based access. In this case, the driver operates as a software interface between the cloud service and the ROS2 network, using the same standardized message definitions.

Although these two acquisition pathways differ in their underlying data access mechanisms, both reconcile sensor data into a unified ROS2 data stream, thereby streamlining integration and simplifying downstream use within the ROS2 ecosystem.

ROS4HC aims to represent human-centered sensing data within the ROS2 network as timely and faithfully as permitted by the underlying sensing devices. ROS4HC drivers are designed to publish measurements immediately upon reception, enabling low-latency and, when supported by the device, waveform-level data.

In practice, the achievable temporal resolution and latency are constrained by the capabilities of the device itself. Some devices allow continuous waveform-level streaming, while others follow a common pattern in which signals are sampled at high rates but transmitted as aggregated data packets at lower frequencies.

For cloud-enabled sensors, real-time access is typically limited, as data must traverse manufacturer-managed systems before it becomes available to the driver. As a result, data may in rare cases be retrieved as a delayed stream, but is more commonly provided in a packetized or batched form, reflecting the data path constraints rather than the framework itself.

Regarding time management, ROS4HC does not impose real-time guarantees nor perform clock synchronization internally, but instead implements low-overhead processing on top of the capabilities exposed by the devices. The framework relies on the time abstraction models provided by ROS2. In single-host deployments, timestamps are typically derived from the local system clock. In distributed multi-host setups, clock synchronization is expected to be handled externally using industry-standard mechanisms such as Precision Time Protocol (PTP) or Network Time Protocol (NTP), which is required for multi-sensor time alignment.

Finally, for devices that internally log measured data, timestamps commonly originate from the device’s own clock. In such cases, maintaining clock accuracy and synchronization is the responsibility of the user. Upon reconnection, ROS4HC preserves the provided timestamps and exposes them to the ROS2 network, enabling offline and delayed data to be integrated consistently with other data streams.

#### 2.3.3 Libraries and utilities

Libraries within the ROS4HC framework are meant to transform actual human sensing data into actionable insights and derived indicators. By leveraging these libraries, developers can harness the full potential of the captured data, enabling intelligent decision-making, and advanced robotic interactions in various settings, from clinical environments to industrial floors. Utilities provide tools and functionalities designed to enhance the usability, connectivity, and interoperability of the framework. These include visualization tools for real-time monitoring and analysis of human data. They are vital for users to interpret data effectively and integrate into broader robotic ecosystems.

**FIGURE 2:**
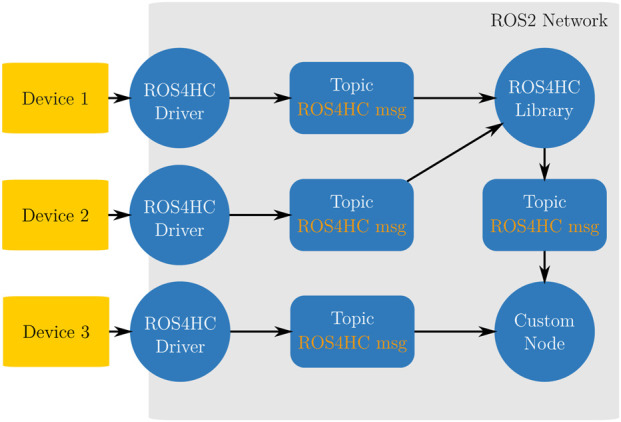
Conceptual diagram of our system, multiple sensors can be brought into the ROS2 network using our drivers and they can then be subscribed directly or a ROS4HC library can be used to process the data and obtain a derived metric. Circles represent nodes and rounded rectangles represent topics, external devices are represented by squares.

A more detailed diagram of the organization of our framework can be seen in Figure 2, here the integration between ROS4HC and client nodes can be seen. Multiple drivers can feed into multiple topics and as such information can either be routed to ROS4HC libraries or utilities to be consumed by client nodes or data can be consumed directly by other nodes in the network.

#### 2.3.4 Comparison against existing frameworks

Our proposed ROS4HC framework provides standardized integration of the human sensing data and provides solutions that are not offered by other methods or State-of-the-Art (SOTA) as explained earlier in subsection 1.1 To explain this, we provide Table 1 where we show the key differences between the current SOTA implementations and our solution.

**TABLE 1:** Comparison of the proposed framework and the currently available solutions.

Framework	Modalities	Applications	Pros	Cons
ROS4HC (Ours)	HR, BR, HRV, EEG, EMG, EOG, mood, gait, CO, SV, BCG, ECG, EDA, ICG, PPG	Robotics, rehabilitation, HRI, safety	ROS 2 native, real-time capable, scalable multimodal integration	Early-stage development, limited device support
Lab streaming layer (LSL)	EEG, ECG, EMG, eye tracking, IMU, MOCAP, biosignals, events, others	Lab synchronization, BCI, multimodal experiments	Widely used, supports many devices, precise time synchronization	No native robotics integration, not ROS2-compatible
Social signal interpretation (SSI)	Audio (speech), video (face and gestures), biosignals	Human–computer interaction, social behavior analysis	Strong multimodal HCI focus, mature signal processing tools	Not designed for robotics or real-time control
ROSNeuro	EEG, EMG	BCI, neuroprosthetics, rehabilitation robotics	ROS-native neural interfaces, standardized messages for BCI data	Limited modality support
ROS4HRI	Face (detection, identification), body (pose, gesture), voice (recognition, identification)	Human–robot interaction	High-level HRI abstractions, easy integration with ROS perception stack	Focused on behavioral cues, no physiological or neural signal support
OpenViBE	EEG, MEG, EMG, others	BCI, neuroscience, neurofeedback	Powerful real-time BCI processing, widely validated in research	Not ROS-native, limited support for robotics integration

## 3 ROS 4 healthcare: initial release

With its first release, ROS4HC introduces new ROS2 drivers for various standard and novel bio-signal acquisition devices, in addition to other sensors that can support better human-robot interaction. This section outlines the main sensor drivers introduced with ROS4HC. For sensors where direct access is technically impossible or permitted, we offer software wrappers to integrate sensor data into the ROS network.

### 3.1 Supported devices

Table 2 provides an overview of the sensors supported in the initial release of ROS4HC, detailing their driver type, modalities, key features, and integration method. This diverse set of sensors demonstrates the framework’s ability to integrate a wide range of human sensing technologies.

**TABLE 2:** Sensor overview and integration in ROS4HC.

Sensor	Driver type	Modalities	Key features	ROS4HC integration	Certified medical device
Sensomative pressure mat	Direct connection; streaming	Distributed pressure sensors	Detects seating posture via pressure distribution. Bluetooth LE comm	Python driver publishes pressure array as custom ROS message	✕
Mbientlab MetaMotionS	Direct connection Streaming	IMU (Acc, Gyr Mag), Temp Light	Wearable multi-sensor With 10-axis IMU. Bluetooth LE comm	Python script parses data using library and publishes to ROS.	✕
Corsano CardioWatch Bracelet	Direct connection Local Storage	PPG, ECG Temp, EDA IMU	Wearable device with local storage. 1 Hz polling rate	ROS publisher for HR BR, and acceleration via Bluetooth LE.	✓
Seeed Studio 60 GHz mm-wave sensor	Direct connection Streaming	HR, BR Movement Sleep	Stationary device 1 Hz polling rate	ROS publisher for HR BR, movement and Waveforms via serial	✕
MAX30100 + M5 StickC PLUS2	Direct connection Streaming	PPG SpO2	Wearable device 1 Hz publishing rate	ROS publisher for HR and SpO2 via WiFi	✕
POLAR H10 Heart rate Sensor	Direct connection Streaming	ECG	Wearable device 1 Hz polling rate	ROS publisher for HR and BR via Bluetooth LE.	✕

A significant challenge in integrating healthcare and wearable sensors is their deviation from traditional robotics sensors. Many rely on cloud-based systems, are subject to contractual restrictions (e.g., Non-Disclosure Agreements or NDAs), or lack sufficient documentation for direct driver development. We address these challenges by providing standardized abstractions to enable their use.

### 3.2 Tools and visualization

The ROS4HC framework provides tools for data analysis and visualization, enabling healthcare professionals to derive meaningful insights from multimodal sensor data in real-time clinical settings.

#### 3.2.1 Activities of daily living (ADL) classifier

The Activities of Daily Living (ADL) metric is crucial for tracking rehabilitation progress, particularly in spinal cord injury patients. Our ADL prediction model classifies activities into 12 categories: Rest, Self Propulsion, Arm Raises, Transfer, Using Phone, Conversation, Washing Hands, Eating/Drinking, Assisted Propulsion, Using Computer, Changing Clothes, and Pressure Relief.

In the paper, it was reported that the optimal classification performance was obtained with the following sensor combination:

1. Wrist accelerometer (Corsano CardioWatch)
2. Ear accelerometer (cmedoalpha)
3. Pressure mat (Sensomative Pressure Mat)
4. Wheel accelerometer and gyroscope (JUMP)

Our current implementation utilizes only one out of the four top performing sensor combination, as well as two additional sensors: Mbient IMU, Vivalink ECG Patch and the Sensomative Pressure Mat. These sensors were chosen based on their availability, low intrusiveness, and ability to provide real-time data.

Despite not using the top performing sensor combination, the selected sensors gave us very satisfactory results. Nevertheless, there was a slight delay of few seconds when changing activities, which is not an alarming caveat in our target application as we are only interested in observing the activities and their duration.

The ADL classifier takes as input the Mbientlab MetaMotionS IMU and Sensomative Pressure Mat data, feeding these points into the model, and gives back a classification result. The model evaluates a predefined data window and returns multiple classification predictions, with the final results determined by majority voting.

#### 3.2.2 Visualization

Effective data visualization is a critical aspect in healthcare applications, as it allows clinicians and researchers to easily interpret multiple visualization tools to display both physiological sensor data and secondary information like ADL classication.

We developed a dashboard using Foxglove Studio Foxglove Technologies Inc. (2024), fully integrated into the ROS4HC ecosystem that visualizes all sensor data and analytical outputs within the ROS4HC framework. The dashboard is organized into two sections: physiological sensor data visualization and secondary data visualization, with the following components:

Physiological Sensor Data

1. Heart and Respiratory Rate Widget: Real-time display of cardiovascular parameters (HR in bpm, respiratory rate in breaths/min) from the Vivalink/Polar sensor.
2. HR History Plot: Displays the variation in heart rate over time. (HR vs. time in seconds)
3. ECG Plot: Plots the ECG readings from the Vivalink sensor (mVolts vs. time in seconds)
4. IMU Plot: Plots acceleration along x, y, and z-axes from the Mbientlab MetaMotionS IMU sensor.
5. Sensomative Pressure Distribution Widget: Heat-map visualization of pressure points from the Sensomative pressure mat placed on the wheelchair cushion, critical for monitoring pressure in wheelchair users.

Secondary Data Visualization

1. ADL Widget: Shows the current daily activity performed by the user.
2. Sleep Pose Widget: Displays the current sleep pose of the user on the Somnomat.
3. ADL History: Displays the time distributed by the user among various ADL classes during the observed time.

### 3.3 Contributing to ROS4HC

We actively encourage developers, researchers, and healthcare technologists to contribute and expand the capabilities of the ROS4HC framework in several ways:

1. Implement new sensor drivers to expand ROS4HC’s hardware compatibility.
2. Add support for both direct connection sensors and cloud-enabled medical devices.
3. Develop tools and algorithms for efficient processing of biosignals data streams.
4. Share use-cases and applications.

For more details and to contribute, download the repository^2^.

## 4 Materials and methods

### 4.1 Materials

All experiments were conducted using the ROS2 middleware (Humble Hawksbill) running on Ubuntu 22.04. The ROS4HC framework was implemented in both Python and C++, following standard ROS2 conventions for message definition and node communication. Data visualization was carried out using Foxglove Studio and logging was done using standard ROSbags.

#### 4.1.1 Sensors

The experiments employed a range of medical and wearable devices (see Table 2), including: Polar H10 (ECG, heart rate), Sensomative Pressure Mat (pressure distribution), and Seeed Studio mm-Wave radar sensor (heart rate, breathing rate, movement). Data rates varied between 1 Hz for PPG/HR and up to 25 Hz for pressure distribution data. Each sensor was connected to the ROS2 network via our custom ROS4HC drivers that published standardized physiological message types.

#### 4.1.2 Robotic platforms

Three robotic systems were used to validate the framework:

1. Heart Rate–Adaptive Wheelchair with Posture-Based Control: A powered wheelchair equipped with a seat-mounted pressure mat and an onboard computer running ROS4HC nodes.
2. AI-enabled personal trainer: A treadmill with speed control integrated through a ROS node, combined with a wearable biosensor and a virtual avatar for user feedback.
3. Nocturnal monitoring system: The Somnomat Care robotic bed, integrating a mm-Wave sensor for non-contact monitoring and actuated rocking/backrest mechanisms for interventions.

### 4.2 Methods

The ROS4HC framework standardizes the integration of human physiological data into robotic systems by defining a unified structure for message types, sensor drivers, and processing libraries. Each sensor publishes data through its respective driver node using custom message definitions (e.g., biosensing/HR, physical/PressureMat). These data streams are processed by library nodes that derive higher-level indicators such as the Center of Pressure (COP). Processed information is then used by control nodes to modulate robot behavior in real-time.

This section provides an overview of the experiments used for testing our framework, further details are provided in the subsequent section.

#### 4.2.1 Heart rate–adaptive wheelchair with posture-based control

In this case study, a ROS2 control node estimated the user’s COP from pressure-sensing cushion data and mapped it to planar velocity commands, representing posture-derived motion intent. In parallel, heart rate measurements acquired from a wearable sensor were incorporated as a physiological modulation signal to adapt the wheelchair’s forward velocity. Specifically, the maximum allowable speed was linearly scaled when the HR exceeded 90 bpm, reaching zero velocity at 125 bpm. Experiments were conducted in a controlled indoor environment using 3-min traversal trials, during which heart rate and commanded velocity signals were logged for offline analysis.

#### 4.2.2 AI-enabled personal trainer

The treadmill system included a ROS node responsible for treadmill speed control and another for biosignal acquisition from the wearable sensor. A dialogue agent (OpenAI ChatGPT 4o-mini) provided adaptive feedback every 30 s, maintaining the user’s HR within a target range of 85–95 bpm, this was done by encouraging the user via voice prompts. Each session lasted 10 min, with three subjects participating. Recorded metrics included treadmill speed, HR time series, and model intervention timestamps.

#### 4.2.3 Nocturnal monitoring and interventions

The Somnomat Care robotic bed employed the Seeed Studio mm-Wave sensor to monitor HR, breathing rate, and movement. The control node adjusted the bed’s rocking period inversely to HR and stopped the motion when excessive movement was detected. When restlessness persisted beyond a threshold, the bed’s backrest was raised to 45 ° to assist user recovery. Each test session lasted 5 min under simulated sleep conditions.

Sensor data was streamed via ROS topics and processed for filtering, windowing, and event detection. All processing parameters and ROS nodes are openly available in the ROS4HC GitHub repository (https://www.github.com/ros-healthcare/ros4healthcare-demos).

## 5 Case studies

To demonstrate how our proposition can be used in real scenarios, in this section we present three showcases of technologies that can be readily implemented. They connect the human state to robots and allow better production of robotic systems which take into account the biosignals of the user and react to their needs.

### 5.1 Heart rate-adaptive wheelchair velocity modulation

To demonstrate the applicability of the ROS4HC framework for physiology-aware human–robot interaction, we conducted a case study using a powered wheelchair with adaptive velocity modulation. The aim was to investigate how real-time HR measurements can be integrated into the control loop to regulate wheelchair velocity according to the user’s physiological state, thereby enhancing operational safety and reducing physical strain. Heart rate is a well-established, non-invasive indicator of cardiovascular workload and physiological stress Schubert et al. (2009); Quigley et al. (2024), and is widely used in ergonomics, rehabilitation, and human–machine interaction research to quantify task-induced physiological demand Mezzani et al. (2013); Sammito et al. (2024). During powered wheelchair operation, elevated HR may arise from increased physical effort, sustained postural demands, environmental complexity, or user anxiety. Persistently elevated HR has been associated with increased perceived exertion, fatigue, and reduced tolerance to physical demands Borg (1998), effects that are particularly pronounced in individuals with mobility impairments or limited physiological reserve van der Woude et al. (2006).

Regulating wheelchair velocity based on HR therefore provides a mechanism to dynamically align system behavior with the user’s momentary physiological capacity. By reducing speed under conditions of elevated cardiovascular load, the controller can lower task demands, increase reaction time, and reduce the risk of unsafe maneuvers, while allowing higher velocities when the user exhibits physiological stability. In this way, HR-based adaptation functions as a safety- and user-centered control signal rather than a direct proxy for intention.

In addition to physical exertion, cardiac measures have been shown to reflect cognitive workload in complex human–robot interaction scenarios Bassi et al. (2025). Consequently, HR-aware velocity modulation may also help accommodate transient increases in mental load, such as those induced by navigation in cluttered or unpredictable environments, further supporting safe and intuitive wheelchair operation.

**FIGURE 3:**
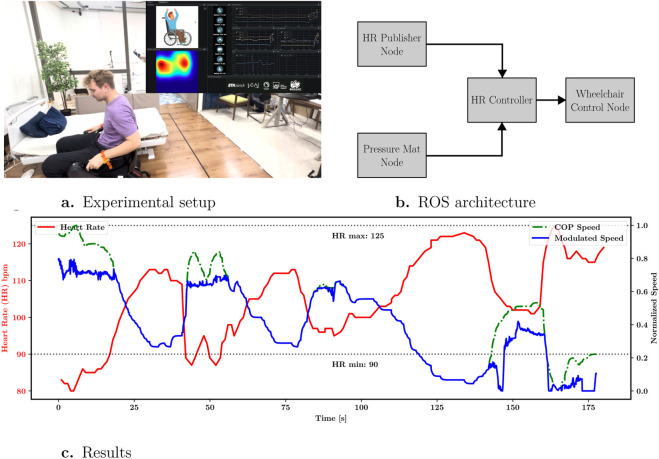
Heart rate–controlled wheelchair traversal. **(a)** Experimental setup. A wearable HR sensor and a pressure-sensing seat mat are used to capture physiological and posture data in real-time. **(b)** Architecture integrating physiological and posture sensing. And the velocity control node responsible for generating movement commands. **(c)** Experimental results demonstrating speed modulation based on HR thresholds. The system modulates forward velocity when HR exceeds 90 bpm, illustrating adaptive physiological control.

Experiments were conducted in a controlled indoor environment which can be seen in Figure 3a using a commercially available powered wheelchair equipped with ROS 2 nodes for physiological sensing and motion control the detail of the node configuration is available in Figure 3b. A wearable HR sensor streamed data at approximately 1 Hz, while a pressure-sensing cushion integrated into the seat provided continuous posture measurements. From the measured pressure distribution, the user’s COP was estimated and mapped to a desired planar velocity command, enabling hands-free wheelchair control through upper-body leaning.

The intent of the user was inferred exclusively from posture. Specifically, lateral COP displacement was mapped to angular velocity, while anterior–posterior displacement was mapped to translational velocity, with dead zones applied to suppress unintended motion. To ensure smooth and safe operation, the resulting velocity commands were subject to proportional–derivative smoothing and explicit acceleration limits.

HR is then used as a physiological modulation signal that regulates the maximum allowable translational velocity of the wheelchair. This formulation better reflects the intended control paradigm: posture expresses voluntary intent, while heart rate constrains the speed at which this intent may be executed. Let $v_{\mathrm{COP}}$ denote the velocity command derived from the COP. The executed command $v_{\mathrm{cmd}}$ is obtained by enforcing a heart-rate-dependent speed limit as seen in Equation 1,

$$v_{\mathrm{cmd}}=\left\{\begin{array}{cc} v_{\mathrm{COP}}, & \|v_{\mathrm{COP}}\|\leq v_{\max}\left(HR\right),\\ \frac{v_{\max}\left(HR\right)}{\left\|v_{\mathrm{COP}}\right\|}\;v_{\mathrm{COP}}, & \|v_{\mathrm{COP}}\|>v_{\max}\left(HR\right). \end{array}\right.$$ (1)

where $v_{\max}\left(HR\right)$ denotes the maximum permitted translational velocity.

In this study, the heart-rate-dependent speed limit was defined as shown in Equation 2

$$v_{\max}\left(HR\right)=\left\{\begin{array}{cc} v_{\max}, & HR\leq {HR}_{\min},\\ v_{\max}\;\left(1-\frac{HR-{HR}_{\min}}{{HR}_{\max}-{HR}_{\min}}\right), & {HR}_{\min}<HR<{HR}_{\max},\\ 0, & HR\geq {HR}_{\max}. \end{array}\right.$$ (2)

with ${HR}_{\min}=90bpm$ and ${HR}_{\max}=125bpm$ . To avoid abrupt changes in behavior due to transient heart-rate fluctuations, the speed limit was further filtered using a proportional–derivative controller before being applied to the velocity command.

The results shown in Figure 3c demonstrate that increases in HR beyond the lower threshold consistently led to a reduction in forward velocity, despite unchanged posture-derived intent. This behavior confirms that voluntary control inputs and involuntary physiological feedback were effectively decoupled and recombined in a principled manner.

Broader Applicability: The proposed control paradigm generalizes to other human-robot collaboration scenarios. For example, industrial robots may modulate speed or enter safe states when physiological indicators such as HR or heart rate variability suggest elevated worker stress or fatigue Sammito et al. (2024), thereby improving safety and ergonomics in shared workspaces Villani et al. (2018).

### 5.2 AI enabled personal trainer based on real-time feedback from biosensors and a social interface

This use case showcases the integration of the presented technologies to implement an AI-enabled personal trainer.

Walking and jogging offer numerous benefits, especially for the elderly, by improving balance, preventing muscle loss, and promoting overall wellbeing. However, outdoor exercise can be risky, making home treadmill workouts a safer option, but it can be monotonous. While personal trainers provide constant guidance and motivation, they are not accessible to everyone, making a virtual personal trainer an effective alternative to encourage proper exercise and maintain motivation.

**FIGURE 4:**
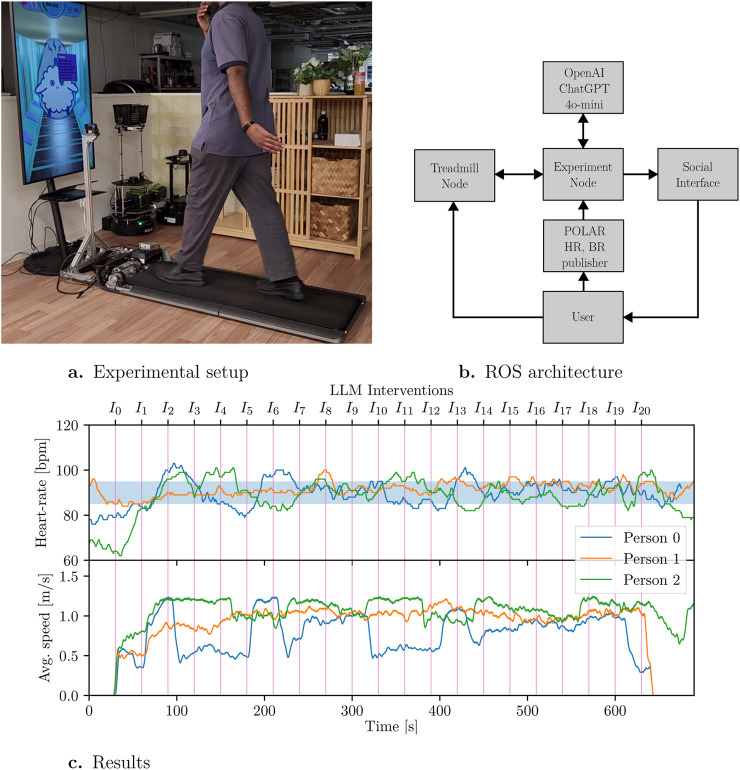
AI-enabled personal trainer. **(a)** Experimental setup. of the AI-enabled personal trainer. The participant walks on a treadmill equipped with a wearable heart rate sensor and an on-screen virtual embodiment providing real-time feedback. **(b)** ROS 2 system architecture of the personal trainer system. Biosensor data from the wearable device and treadmill are processed by ROS4HC nodes, which interact with an LLM-based dialogue agent to generate adaptive exercise instructions. **(c)** Heart rate and treadmill speed profiles for each participant. The vertical red lines indicate interventions generated by the LLM every 30 s, and the light blue shaded area denotes the target heart rate range (85–95 bpm).

Our proposed framework, ROS4HC, enables the development of such a system by seamlessly integrating biosensor data and exercise monitoring as can be seen in Figure 4b. We applied ROS4HC to connect an adaptive treadmill that adjusts its speed based on the actual walking speed of the user, a wearable band for HR and breathing rate monitoring, and a virtual embodiment for user interaction, the experimental setup is presented in Figure 4a.

Before starting, the user chooses the session duration and intensity for their workout. A user interface displays real-time data during the session, including speed, distance, vital signs, and the remaining time. These parameters are continuously monitored by a ROS4HC-based node, which evaluates the user’s current heart rate relative to a predefined target range (85–95 bpm). Based on this comparison, the system periodically generates adaptive, natural-language feedback via OpenAI’s LLM (ChatGPT-4o-mini), encouraging the user to increase or decrease walking speed as needed to return toward the target physiological zone. The feedback is delivered through a virtual embodied agent displayed on the screen, serving as an intuitive and non-intrusive coaching interface rather than a direct actuator. To test the system, three experiments were conducted, for each of the participants, a plot of their heart rate and the treadmill speed can be seen in Figure 4c, the vertical purple lines represent the interventions by the LLM, which occur every 30 s, and the light blue hue represents the target heart rate range.

Experiments showed that participants stayed within the target range for most of the session using only verbal instructions. The treadmill’s speed limit (1.3 m/s) restricted higher heart rates, but the results demonstrate the potential of this approach for enhancing engagement and motivation in home-based exercise.

### 5.3 Nocturnal monitoring and interventions

Sleep apnea is among the most common sleep-related breathing disorders in adults and includes several subtypes, with obstructive sleep apnea (Obstructive Sleep Apnea (OSA)) being the most prevalent. OSA arises when the throat muscles relax excessively during sleep, causing interruptions in breathing. In this use case, we envision a plausible intervention in which the user’s movements and heart rate are continuously monitored to help prevent such episodes. To realize this concept, we employed the Somnomat Care robotic bed Manriquez-Cisterna et al. (2025), developed as part of the long-standing Somnomat project. This bed can gently rock the user to promote more stable sleep patterns. Previous research Ravesloot et al. (2017); Meszaros et al. (2022); Breuss et al. (2024) indicates that approximately 56%–75% of apnea cases are positional in nature, motivating the development of such interventions aimed at mitigating position-dependent sleep apnea.

#### 5.3.1 Experiment description

**FIGURE 5:**
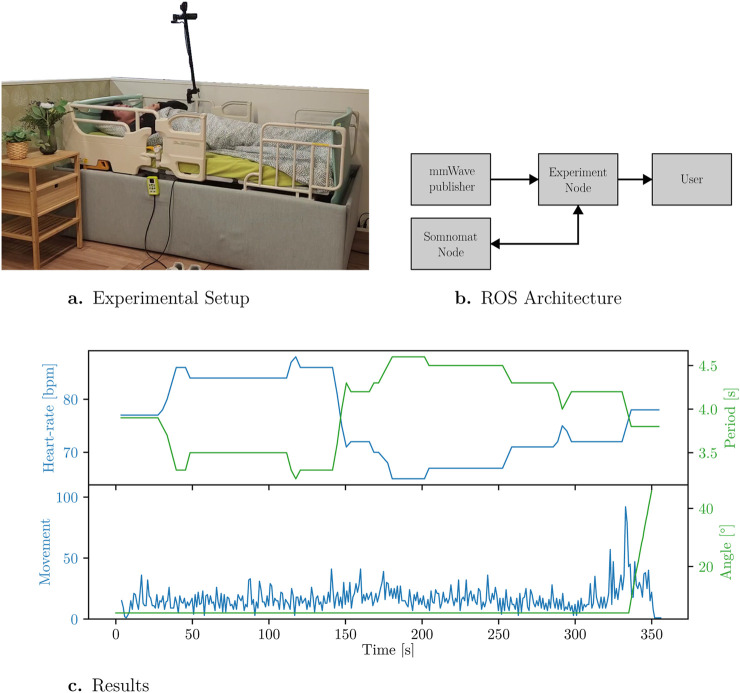
Nocturnal monitoring and intervention. **(a)** Experimental setup of the Somnomat Care robotic bed. The system includes integrated sensors for monitoring heart rate, breathing rate, and movement during simulated sleep conditions. **(b)** ROS 2 architecture of the Somnomat Care system. The mmWave sensor data streams (HR, BR, and movement) are processed in real-time to control the bed’s rocking and backrest mechanisms. **(c)** Time series of heart rate, rocking period, and movement levels. The upper plot shows how increases in heart rate trigger faster rocking, while the lower plot indicates how elevated movement levels stop rocking and lift the backrest to 45° for user recovery.

In this experiment, a subject remained awake while lying on the bed for 5 min. During this period, a mm-Wave sensor monitored their HR, breathing rate (BR), and movement (for this sensor, movement is a metric ranging from 0 to 100, providing a relative indication of activity), the experimental setup can be seen in Figure 5a. To demonstrate the intervention, an algorithm was implemented that links the subject’s heart rate to the speed of the bed’s rocking motion. Specifically, increase in heart rate following an apnea episode trigger a faster rocking speed, encouraging the user to adjust their position. If the user becomes too restless or uncomfortable, a movement detection algorithm halts the rocking motion and raises the backrest to assist the user in waking up and returning to a calm state.

Figure 5b shows the ROS node connections for this open-loop intervention. Figure 5c illustrates the relationship between heart rate and the bed’s rocking period. The lower part of the figure shows movement detection and how it triggers the stopping of the rocking motion and the lifting of the backrest.

#### 5.3.2 Determination of an apnea episode

Sleep apnea usually presents itself with a drop in heart rate (bradycardia), followed by a sudden increase (tachycardia) when the episode ends. In this showcase, a mm-Wave sensor captures these physiological changes and feeds them into a positive feedback loop linked to the bed’s rocking motion. For example, the tachycardia following an apnea episode triggers an increase in the rocking speed, which in turn induces subtle movement in the user, encouraging repositioning and promoting comfort.

#### 5.3.3 Stress and restlessness categorization

Using a moving window average, the mean movement as measured by the mm-Wave sensor is evaluated. This approach prevents brief, isolated movements from triggering the intervention, while ensuring that sustained restlessness activates it. When triggered, the intervention stops the bed’s rocking motion and raises the backrest to 45deg , which typically awakens the subject and allows them to regain control and relax after the apnea episode.

Broader Applicability: The principles of continuous, unobtrusive human monitoring and adaptive physical intervention have relevance beyond sleep care. For instance, in control rooms or remote operation centers, ROS4HC could monitor operator alertness or physiological distress using mm-Wave sensors or wearable devices. If critical thresholds are exceeded, an automated system could trigger alarms, initiate communication, or even take over non-critical tasks to maintain safety and prevent human error.

In this way we have shown how our framework can be applied to real examples where seamless integration with human-state and robot interventions can be realized. Based on this information, we believe other related human-robot interaction application development can be accelerated by using this framework.

## 6 Discussion

In this section, we explore the implications of the initial release of ROS4HC and the user case studies, which demonstrate the practical applications of the ROS4HC framework in real-world applications to enhance human-robot interaction and safety.

The heart-rate controlled wheelchair case study demonstrates the potential of integrating real-time physiological data into robotic control systems as a novel approach to human-robot interaction. Beyond just adjusting wheelchair speed, this approach represents a shift in how robotic systems can interpret and respond to users’ physiological states. By modulating velocity based on heart rate, the system creates a responsive, adaptive interface that prioritizes user safety and comfort. This example serves as a proof-of-concept for physiological signal integration, illustrating how biosignals can transform robotic assistive technologies from static, rigid systems to intelligent, context-aware platforms that can intuitively adjust to a user’s momentary physiological condition. On the other hand, the AI-enabled personal trainer used biosensor data, wearable devices, and AI algorithms to create personalized workouts for elderly users. By maintaining participants within a target heart rate range and using AI-driven feedback, the system served both as a motivational tool and a means of safely guiding exercise intensity. The results illustrate how embedding physiological information into the control loop can enhance engagement and promote effective home-based exercise.

Similarly, the nocturnal monitoring system for sleep apnea highlights the value of real-time biosignal tracking of health conditions. By combining heart rate and movement data, the robotic bed was able to identify sleep apnea episodes and trigger interventions, thereby improving sleep quality and reducing the risks associated with apnea.

These demonstrators also highlight the importance of collaboration between different technological domains, including robotics, AI, and healthcare, to develop comprehensive solutions for future healthcare challenges. Moreover, the results from these case studies show the potential of integrating biosignals into robotic systems. By allowing robots to adapt their behavior based on real-time physiological data, healthcare providers can offer more personalized and responsive care solutions. Likewise, roboticists can integrate the same technologies to enhance safety and adaptability in a variety of robotic systems. The presented framework is broadly extensible, with applications in healthcare ranging from elderly care and rehabilitation to chronic disease management and sleep disorder treatment. Beyond healthcare, these principles offer numerous opportunities in human–robot interaction and safety, potentially improving the reach and effectiveness of robotic systems across multiple domains.

### 6.1 Future directions

Future work will focus on expanding the range of supported sensors and integrating more advanced human state estimation algorithms within our libraries. Furthermore, fostering community contributions and advocating for ROS-compatible interfaces with device manufacturers will be key to the continued growth and impact of ROS4HC in advancing human-centric robotics.

Future iterations of ROS4HC should prioritize integration with mobile health (mHealth) technologies and compliance with relevant international standards. Specifically, aligning the framework with ISO/IEEE 11073 standards for personal health device communication would ensure robust interoperability with certified medical devices and healthcare information systems. Implementation of ISO/TS 82304-2:2021 quality criteria for health software would strengthen the framework’s reliability, security, and usability in clinical settings. Such standards compliance would not only strengthen the technical foundation of ROS4HC but also accelerate regulatory approval processes and promote its adoption as a trustworthy platform for next-generation healthcare robotics solutions.

## 7 Conclusion

This paper introduces ROS for Healthcare (ROS4HC), a standardized open-source ROS2 framework that addresses the critical need for robust and generalized human sensing integration in robotics. By providing unified message types, device drivers, processing libraries, and visualization utilities, it simplifies the complex task of incorporating physiological, emotional, and physical human signals into robotic systems. This framework significantly reduces development time and enhances system interoperability, particularly for medical devices and wearables that traditionally lack native ROS support.

ROS4HC benefits researchers and industry by providing a modular platform for biosignal processing, medical applications, assistive robotics, social robotics and industrial safety related applications. It standardizes medical device integration, reducing development time and creating system interoperability. Additionally, it promotes collaboration through open-source contributions and advocacy for ROS-compatible medical devices.

We have demonstrated the practical utility and real-world deployment through empirical case studies. These examples showcase how the framework enables robots to adapt intelligently to human states, leading to more responsive, safe, and personalized interactions. Crucially, we have highlighted our broad applicability beyond healthcare, extending our relevance to domains such as industrial safety, human-robot collaboration, and performance monitoring. The framework’s design principles—modularity, abstraction, and extensibility—make it a versatile tool for any application requiring sophisticated human state awareness.

## Data Availability

Publicly available datasets were analyzed in this study. This data can be found here: https://www.github.com/ros-healthcare/ros4healthcare-demos.

## Footnotes

1
https://www8.cao.go.jp/cstp/english/moonshot/sub3_en.html

2
https://www.github.com/ros-healthcare/ros4healthcare
