# Correction: ROS 4 healthcare: a framework for physiological human sensing for social, assistive, rehabilitation, and medical robotics

**DOI:** 10.3389/frobt.2026.1854816

**Published:** 2026-05-18

**Authors:** Ricardo Javier Manríquez-Cisterna, Pranjal Mishra, Jorge Peña-Queralta, Monica Perez-Serrano, Spyridon Garyfallidis, Lucas Kupper, Mehdi Ejtehadi, Alexander Breuss, Ankit A. Ravankar, Jose Victorio Salazar Luces, Robert Riener, Yasuhisa Hirata, Diego Paez-Granados

**Affiliations:** 1 Smart Robots Design Lab, Department of Robotics, Graduate School of Engineering, Tohoku University, Sendai, Miyagi, Japan; 2 Spinal Cord Injury and Artificial Intelligence Lab, Department of Health Science and Technology, ETH Zurich, Zurich, Switzerland; 3 Digital Healthcare and Rehabilitation, Swiss Paraplegic Research, Nottwil, Switzerland; 4 Sensory-Motor Systems Lab, Institute of Robotics and Intelligent Systems, ETH Zurich, Zurich, Switzerland; 5 Spinal Cord Injury Center, University Hospital Balgrist, Zurich, Switzerland

**Keywords:** biosignals, healthcare robotics, human-robot interaction, medical robotics, open-source, rehabilitation robotics, robot operating system, social robotics

The reference Kruse, T., Pandey, A. K., Alami, R., and Kirsch, A. (2013) Human-aware robot navigation: A survey. *Robot. Auton. Syst.* 61(12), 1726-1743. doi: 10.1016/j.robot.2013.05.007 was erroneously given as Lemaignan, S., Alami, R., and Fiore, M. (2024). Human-aware robotics: a survey of state, challenges, and opportunities. IEEE Robotics Automation Mag. 31, 26–39. doi: 10.1109/MRA.2023.3330124

The reference Quigley, K. S., Gianaros, P. J., Norman, G. J., Jennings, J. R., Berntson, G. G., de Geus, E. J. C. (2024). Publication guidelines for human heart rate and heart rate variability studies in psychophysiology–Part 1: Physiological underpinnings and foundations of measurement. *Psychophysiology* 61, e14604. doi: 10.1111/psyp.14604 was erroneously given as Quintana, D. S., Alvares, G. A., and Heathers, J. A. J. (2024). Publication guidelines for human heart rate and heart rate variability studies in psychophysiology–part 1: physiological underpinnings and foundations of measurement. *Psychophysiology*. doi: 10.1111/psyp.14625.

The reference Estrin, D., and Sim, I. (2010) Open mHealth architecture: an engine for health care innovation. *Science* 330, 759–760. doi: 10.1126/science.1196187 was erroneously given as Open mHealth (2023). Open mHealth: an open standard for mobile health data.

Author Jorge Peña-Queralta was erroneously assigned to affiliation Smart Robots Design Lab, Department of Robotics, Graduate School of Engineering, Tohoku University, Sendai, Miyagi, Japan. This affiliation has now been removed for author Jorge Peña-Queralta.

The present address Centre for Artificial Intelligence, Zurich University of Applied Sciences, Winterthur, Switzerland for author Jorge Peña-Queralta as erroneously given as Zurich University of Applied Sciences, Winterthur, Switzerland.

The original article has been updated.

